# Myocardial transfection of hypoxia-inducible factor-1α and co-transplantation of mesenchymal stem cells enhance cardiac repair in rats with experimental myocardial infarction

**DOI:** 10.1186/scrt410

**Published:** 2014-02-07

**Authors:** Bingqing Huang, Juying Qian, Jianying Ma, Zheyong Huang, Yunli Shen, Xueying Chen, Aijun Sun, Junbo Ge, Haozhu Chen

**Affiliations:** 1 Department of Cardiology, Shanghai Institute of Cardiovascular Diseases, Zhongshan Hospital, Fudan University, 180 Feng Lin Road, Shanghai 200032, China

## Abstract

**Introduction:**

Mesenchymal stem cells (MSCs) have potential for the treatment of myocardial infarction. However, several meta-analyses revealed that the outcome of stem cell transplantation is dissatisfactory. A series of studies demonstrated that the combination of cell and gene therapy was a promising strategy to enhance therapeutic efficiency. The aim of this research is to investigate whether and how the combination of overexpression of hypoxia-inducible factor-1α (HIF-1α) and co-transplantation of mesenchymal stem cells can enhance cardiac repair in myocardial infarction.

**Methods:**

We investigated the therapeutic effects of myocardial transfection of HIF-1α and co-transplantation of MSCs on cardiac repair in myocardial infarction by using myocardial transfection of HIF-1α via an adenoviral vector. Myocardial infarction was produced by coronary ligation in Sprague-Dawley (SD) rats. Animals were divided randomly into six groups: (1) HIF-1α + MSCs group: Ad-HIF-1α (6 × 10^9^ plate forming unit) and MSCs (1 × 10^6^) were intramyocardially injected into the border zone simultaneously; (2) HIF-1α group: Ad-HIF-1α (6 × 10^9^ plate forming unit) was injected into the border zone; (3) HIF-1α-MSCs group: Ad-HIF-1α transfected MSCs (1 × 10^6^) were injected into the border zone; (4) MSCs group: MSCs (1 × 10^6^) were injected into the border zone; (5) Control group: same volume of DMEM was injected; (6) SHAM group. Cardiac performance was then quantified by echocardiography as well as molecular and pathologic analysis of heart samples in the peri-infarcted region and the infarcted region at serial time points. The survival and engraftment of transplanted MSCs were also assessed.

**Results:**

Myocardial transfection of HIF-1α combined with MSC transplantation in the peri-infarcted region improved cardiac function four weeks after myocardial infarction. Significant increases in vascular endothelial growth factor (VEGF) and stromal cell-derived factor-1α (SDF-1α) expression, angiogenesis and MSC engraftment, as well as decreased cardiomyocyte apoptosis in peri-infarcted regions in the hearts of the HIF-1α + MSCs group were detected compared to the MSCs group and Control group.

**Conclusions:**

These findings suggest that myocardial transfection of HIF-1α and co-transplantation of mesenchymal stem cells enhance cardiac repair in myocardial infarction, indicating the feasibility and preliminary safety of a combination of myocardial transfection of HIF-1α and MSC transplantation to treat myocardial infarction.

## Introduction

Despite substantial therapeutic advances over the past decade, heart failure, due in large part to myocardial infarction (MI), remains a leading cause of morbidity and mortality worldwide. Stem cell transplantation, as a promising therapy for patients suffering from myocardial infarction, has recently been a research priority. It has huge potential for cardiac regeneration and cardiac function recovery. However, several meta-analyses revealed that the outcome of stem cell transplantation is dissatisfactory. Three to six months after the transplantation of bone marrow-derived stem cells, left ventricular ejection fraction (LVEF) was improved by merely 2.53 to 3.66%, and major adverse cardiac events (MACE) were not significantly decreased [1-3]. The low homing rate and local survival rate of the transplanted cells, affected by endogenous and environmental factors in the ischemic tissue, such as hypoxia, oxidative stress and inflammation, which may result in apoptosis of transplanted cells [4-6], restrain the application of this technique. Strategies to improve cardiac homing and engraftment of stem cells may improve the outcome of this approach [7-12]. One interesting strategy is the combination of cell and gene therapy [13-16]. Satoshi Sintani *et al*. reported that combined intramyocardial CD34^+^ cells and *VEFG2* gene therapy after MI results in better therapeutic effect than monotherapy, though the improvement of cardiac function is still not satisfactory [17].

Hypoxia-inducible factor-1α (HIF-1α) is a major regulator of the hypoxic response after myocardial infarction [16]. Decreased tissue oxygen causes nuclear accumulation of HIF-1α protein and enhancement of its transcriptional activity through binding to enhancer elements in target genes, including *vascular endothelial growth factor (VEGF)*[18], *angiopoietin-1 (Ang-1)*, *angiopoietin-2 (Ang-2)*, *platelet-derived growth factor beta*[19], *nitric oxide synthase (iNOS)*[20], *erythropoietin*[21], *phosphoglycerate kinase*[22] and *stromal-derived factor-1(SDF-1)*. Specifically, the up-regulation of CXCR4 expression in mesenchymal stem cells (MSCs) mediates a broad range of biological processes including cell proliferation, survival, migration, adhesion, differentiation, as well as pro-angiogenesis [23-27].

Therefore, HIF-1α regulates adaptation to hypoxia at the systemic, tissue and cellular levels [28,29], including enabling transcriptional activation of angiogenesis genes, improving the recruitment of endothelial progenitor cells to areas of tissue ischemia through an SDF-1-CXCR4 pathway [30], and affecting the activation of pro-inflammatory chemokine production by endothelium through transcriptional modulation of heme oxygenase-1 [31]. Therefore, *HIF-1α* could be an ideal candidate to improve cell-mediated cardiac repair. Recently, Inmaculada Cerrada *et al*. [32] used *HIF-1α*-transfected MSC transplantation in a rat model of MI, and improvement was observed in terms of cardiac function, angiogenesis, cardiomyocyte proliferation and reduction of fibrotic tissue. It demonstrated that *HIF-1α* gene therapy can enhance cell-mediated therapy for cardiac regeneration. However, the expression level of *HIF-1α* in the ischemic area was not quantified in their study. Since the survival and engraftment of intramyocardial MSC transplantation were less than 5% at two weeks after transplantation [4-6], it is reasonable to deduce that the expression level of HIF-1α from survived *HIF-1α*-transfected MSCs (HIF-1α-MSC) in ischemic myocardium is very low, and enhanced *HIF-1α* expression may lead to better results. Moreover, previous study demonstrated that exogeneous expression of *HIF-1α* by using transfection is significantly higher compared to the endogenous *HIF-1α* expression [29,33]. Thus, in order to get higher expression of *HIF-1α* in the ischemic area instead of just the transplanted cells, we chose to use intramyocardial transfection of *HIF-1α*.

Accordingly, we plan to test the hypothesis that exogeneous expression of *HIF-1α* in the ischemic area may increase the local survival and engraftment of the transplanted MSCs, enhance the angiogenesis, and improve cardiac performance in rats after myocardial infarction. Furthermore, we try to investigate whether intramyocardial transfection of *HIF-1α* and co-transplantation of MSC transplantation has a better capacity of cardiac repair than the transplantation of *HIF-1α*-transfected MSCs.

## Materials and methods

This study conformed to the guiding principles for the Care and Use of Laboratory Animals published by the US National Institutes of Health (NIH Publication No. 85–23, revised 1996). All animal protocols were approved by the Institutional Animal Care and Use Committee of Zhongshan Hospital, Fudan University, PR China. All surgeries were performed under ketamine anesthesia, and all efforts were made to minimize suffering.

### Isolation, culture and transfection of MSCs

Isolation and culture of MSCs were performed as previously described [34]. In brief, MSCs were harvested from the femur and tibia of male Sprague-Dawley rats with a body weight of 80 to 100 g, which were obtained from the Experimental Animal Center of Fudan University (Shanghai, China). Bone marrow cells were flushed and cultured with Iscove’s Modified Dulbecco’s Medium (Invitrogen/Gibco, Frederick, MD, USA) supplemented with 20% fetal bovine serum (FBS) and penicillin (100 U/ml)/streptomycin (100 μg/ml) at 37°C in humid air with 5% CO_2_. After being seeded for two days, MSCs adhered to the bottom of the culture plates, and the non-adherent cells were removed by a medium change at 48 hours. At 80% confluence, cells were harvested with 0.25% trypsin and passaged at a ratio of 1:3. The MSCs (P4) were identified with antibodies against CD105, CD166, CD45 and CD34 (fluorescein isothiocyanate conjugated, FITC).

MSCs were transfected with *Ad-null-green fluorescent protein (Ad-null-GFP)* or *Ad-HIF-1α-GFP* (Genechem Co. Ltd., Shanghai, China.) for 7 h followed by maintenance in the viral vector-free DMEM for 72 h. The successful transduction was judged by the presence of green fluorescence. Randomly selected microscopic fields (n >8; 400×) at 72 h after transduction were evaluated to calculate the ratio of green cells to the total number of cells. These cells were further confirmed by immunostaining for HIF-1α in either *Ad-null-GFP* transfected MSCs (^Ad-Null^MSCs) or *Ad-HIF-1α-GFP* transfected MSCs (^Ad-HIF-1α^MSCs).

### Assessment of MSCs viability and proliferation

A Cell Counting Kit-8 (CCK-8) (Sigma-aldrich, St. Louis, MO, USA) based colorimetric assay was used to quantify cell proliferation. The assay was performed at 12 h, 24 h, 36 h and 48 h on MSCs, ^Ad-Null^MSCs and ^Ad-HIF-1α^MSCs. All MSCs were incubated with 10 μl of CCK-8 tetrazolium salt for 2 h, and the absorbance was read using a microplate spectrofluorometer at a 450-nm wavelength. The number of living cells is directly proportional to the amount of formazan dye that can only be produced by viable cells and generated by the activity of dehydrogenase. The proliferation experiments were repeated three times with each condition tested in triplicate.

### MSCs migration assay

To study the effect of HIF-1α on MSC migration, 10,000 cells treated with or without *HIF-1α* transfection were seeded in the top chamber of an 8 mm-pore migration transwell (Corning, Inc. New York, NY, USA), supplemented with 600 μl DMEM culture medium containing 0.5% FBS in the bottom chamber. After incubation for 12 hours, the inside of the transwells were wrapped with a cotton bud to remove non-migrating cells, the membrane was cut and placed on a glass slide with the bottom side upward, and 4, 6-diamino-2-phenylindole (DAPI) (Life Technologies Corporation, Carlsbad, CA, USA.) was added to stain the nuclei and the migrated cells were counted. The assay was performed in duplicated wells and repeated three times.

### Cell labeling

MSCs were stained by using 10 μg/mL 1,1-dioctadecyl-3,3,3,3-tetramethyl indotricarbocyanine Iodide (DiR, ABD Bioquest, Inc., Sunnyvale, CA, USA) as previously described [35-37]. Cells were then resuspended in growth media at a density of 25 × 10^6^ cells/mL and then kept on ice before transplantation.

### Animal model of myocardial infarction

The experimental animals used in this study were eight-week-old female SD rats. Rats were intraperitoneally anesthetized with ketamine (15 to 20 mg/kg). A midline anterior cervical skin incision was made and the trachea was exposed by sharp dissection. The trachea was intubated with an angiocatheter and ventilated to a rodent ventilator with room air. A 1.5 cm vertical left parasternal skin incision was made, the chest cavity was entered through the fourth interspace, and the pericardium was vertically opened. The left anterior descending coronary artery (LAD) was ligated with a 6-0 polypropylene suture. Ventricle blanching indicated successful occlusion of the vessel. Sham-operated animals served as surgical controls and were subjected to the same procedures as the experimental animals with the exception that the LAD was not ligated. Mortality rates during and after surgery were less than 5% in all groups.

### Implantation of MSCs and Ad-HIF-1α transfection

Female adult SD rats (n = 180) were randomly divided into six groups. Immediately after ligation of the LAD, the HIF-1α + MSCs group (n = 30) received 1 × 10^6^ MSCs and 6 × 10^12^ plate forming unit (PFU) *Ad-HIF-1α* resuspended with 40 ul DMEM, respectively; the HIF-1α group (n = 30) received 6 × 10^12^ PFU *Ad-HIF-1α* and 40 ul DMEM; the HIF-1α-MSCs group (n = 30) received 1 × 10^6 Ad-HIF-1α^MSCs and 6 × 10^12^ PFU *Ad-null* (Genechem Co. Ltd, Shanghai, China); the MSCs group (n = 30) received 1 × 10^6^^Ad-Null^MSCs and 6 × 10^12^ PFU *Ad-null*; and the Control group (n = 30) received 6 × 10^12^ PFU Ad-null and 40 ul DMEM; the SHAM group (n = 30) was subjected to the same procedure as the experimental animals with the exception that the LAD was not ligated. Cells and adenovirus were directly injected into the ischemic border zone of the myocardium at four different sites (20 μl to each site).

### Immunostaining

For HIF-1α staining, MSCs were fixed for 30 minutes with 4% paraformaldehyde and permeabilized for 10 minutes with 0.2% Triton X-100. Cells were blocked with PBS containing 10% FBS overnight at 4°C and incubated with mouse monoclonal anti-HIF-1α antibody (Abcam Biochemicals, Cambridge, UK) at a dilution of 1:100. After thoroughly washing, the cells were incubated with secondary antibodies of goat anti-mouse IgG conjugated with rhodamine (Sigma-aldrich, St. Louis, MO, USA) at a dilution of 1:500 for 1 h at room temperature. Nuclei were stained with DAPI.

Opti-mum cutting temperature compound (O.C.T compound) embedded hearts were sectioned into 5 μm slices. Adjacent sections (taken at the midpoint between LAD ligation site and apex) were double stained with antibodies against rat CD31 (Abcam Biochemicals, Cambridge, UK) and α-SA (α-sarcomeric actinin) (Abcam Biochemicals, Cambridge, UK). Capillary density was defined as CD31^+^ endothelial cells per high-power field (200×). Five high-powered fields were counted per section, with 10 sections/heart, and 10 hearts/group. Angiogenesis in the infarction/peri-infarcted regions was confined to vessels measuring less than 200 μm in diameter.

Apoptosis of cardiomyocytes in the border zone was detected by terminal deoxynucleotidyl transferase dUTP nick end labeling (TUNEL) staining (Roche, Mannheim, Germany) at seven days after the surgical procedure. The number of TUNEL-positive nuclei and the total number of nuclei in three different fields (×400 magnification) were counted by a blinded rater in the border zone (n = 5 in each group). Cardiomyocyte apoptosis is expressed as the ratio of TUNEL-positive nuclei to the total number of cardiomyocyte nuclei.

### Echocardiography

Transthoracic echocardiography (VEVO 770™-230, VisualSonic, Seattle, WA, USA) was performed at one week, two weeks and four weeks post-infarction in each group. LVEF and fractional shortening (FS) were measured as previously described [38]. All measurements were averaged for three consecutive cardiac cycles.

### RT-PCR

The total RNA was isolated from the peri-infarcted myocardial tissues at one week, two weeks and four weeks after surgery (n = 4 in each group at each time point) using TRIzol reagent (Life Technologies Corporation, Carlsbad, CA, USA). The mRNA levels of *HIF-1α*, *SDF-1α*, *VEGF* were determined by using RT-PCR. The primer sequences of *HIF-1α* ([GenBank: NM_024359.1]), *SDF-1α* ([GenBank: NM_001033882.1]), *VEGF* ([GenBank: NM_ 001110333.1]) and *β-actin* ([GenBank: NM_031144.3]) were shown in Table 1. PCR conditions were: 40 cycles of denaturation at 95°C for 30 seconds, annealing at 60°C for 30 seconds and extension at 72°C for 30 seconds. The PCR products were subject to electrophoresis on 1.5% agarose gels, scanned and semi-quantitated by using Image-Quant software (Kodak 1D V3.53).

**Table 1:** Sequence of the primers used in the study

**Gene symbol**	**Primer sequence**	**Product size (base pair)**
*HIF-1α*	Sense: 5′- ATGTGACCATGAGGAAATGAGAGAA-3′	186
	Antisense: 5′- ACGTGAATGTGGCCTGTGCA -3′	
*VEGF*	Sense: 5′-TGCACCCACGACAGAAGGGGA-3′	364
	Antisense: 5′-TCACCGCCTTGGCTTGTCACAT-3′	
*SDF-1α*	Sense: 5′-AGATGCCCCTGCCGATTCTTTG-3′	118
	Antisense: 5′-TGTTGTTGCTTTTCAGCCTTGC-3′	
*β-actin*	Sense: 5′-TCAGGTCATCACTATCGGCAAT-3′	432
	Antisense: 5′-AAAGAAAGGGTGTAAAACGCA-3′	
*SRY*	Sense: 5′-TCTGCTCCTACCTATGCCAACA-3′	22
	Antisense: 5′-GAGGGAACTCAGTATCCAAACCA-3′	

### Quantification of engraftment by real-time PCR

MSCs isolated from male SD rats were injected into female rats, enabling detection of the *SRY* gene (located on the Y chromosome ([GenBank: FJ168067.1]) as an index of engraftment. Quantitative RT-PCR was performed at one week and three weeks after injection (n = 5 for each group). The whole heart was harvested, weighed and homogenized. Genomic DNA was extracted by using the Purelink genomic DNA kit (Life Technologies Corporation, Carlsbad, CA, USA) and quantified with the Quant-it DSDNA assay kit. Triplicate real-time PCR reactions were performed using 50 ng of the genomic DNA. The real-time PCR conditions consisted of an initial denaturation step of 10 minutes at 95°C, followed by 40 cycles at 95°C, for 15 sec, and at 58°C, for 1 minute. A standard curve was generated with multiple dilutions of genomic DNA isolated from male hearts to quantify the absolute gene copy numbers.

### Fluorescence imaging

Fluorescence imaging was performed at three weeks after cell injection (n = 5 for each group). Extensive PBS washing was performed to remove cells adherent to the epicardium. Hearts were placed in a Carestream In-Vivo Multispectral Imaging System FX PRO (Carestream Health, Inc., Rochester, NY, USA) to detect DiR fluorescence under 748 nm of excitation and 780 nm of emission. The exposure time was set at 3 sec and was maintained during the entire imaging session. Hearts from the Control group were also imaged to normalize the noise from background. The fluorescent intensity was calculated using Kodak MI software 5.0.1. Fluorescence signals (photon/s/mm^2^) from a fixed region of interest (ROI) were measured as previously described [39].

### Statistics

Continuous variables with normal distribution were expressed as average ± standard deviation and compared by using Holm’s *t*-test or variance analysis (ANOVA). Categorical variables were expressed as frequencies and percentages. For comparisons between different groups, the chi-square test or Fisher’s exact test was used. A bi-caudal value of *P* <0.05 was considered as statistically significant.

## Results

### Transfection efficiency of Ad-null-GFP and Ad-HIF-1α-GFP in MSCs

**Figure 1:**
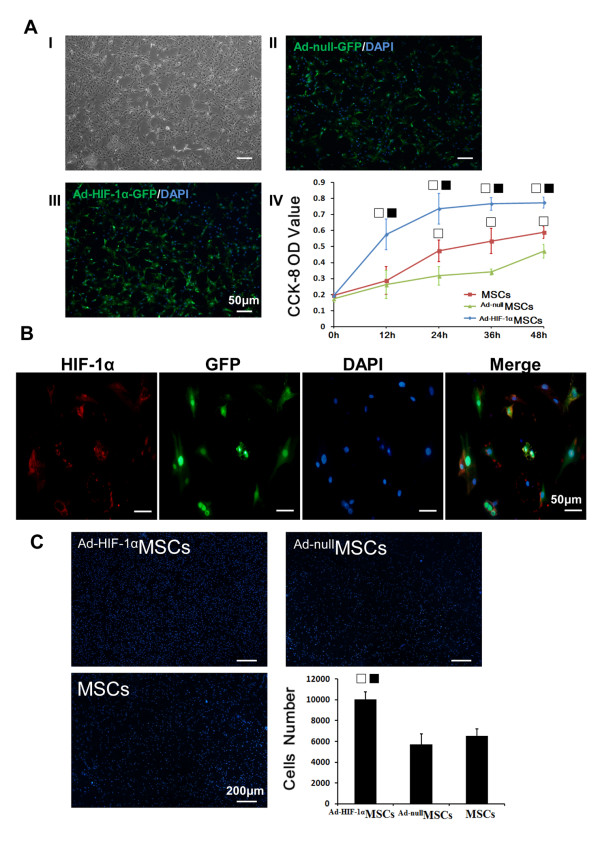
**Effects of HIF-1α on MSCs *in vitro*. A)** Hypoxia-inducible factor-1α (HIF-1α) improved the viability and proliferation of mesenchymal stem cells (MSCs). (I) Phase contrast image of MSCs, (II) ^Ad-null^MSCs, (III) ^Ad-HIF-1α^MSCs, (IV) A Cell Counting Kit-8-based colorimetric assay to quantify the MSCs viability and proliferation. **B)** MSCs were stained with anti-HIF-1α antibody (red) and 4, 6-diamino-2-phenylindole (DAPI) (blue). No HIF-1α was detected in MSCs group and ^Ad-null^MSCs. **C)** MSC migration assay showed HIF-1α improved the motility of MSCs. □*P* <0.01 vs. ^Ad-null^MSCs, ■*P* <0.01 vs. MSCs.

Immunostaining showed that the isolated MSCs (P4) (Figure 1A I) uniformly expressed CD105 and CD166 (see Additional file 1A), but not CD45 or CD34. The differentiation assay confirmed the differentiation potentials of the isolated MSCs into osteoblasts, adipocytes and chondrocytes (see Additional file 1B). The MSCs with a fusiform shape were distributed uniformly at 24 h after transfection with *Ad-null-GFP*. (Figure 1A II) or *Ad-HIF-1α-GFP* (Figure 1A III), and the majority of the cells (94 ± 3.5% and 93 ± 3.8%, respectively) expressed GFP. Immunofluorescence confirmed the expression of *HIF-1α* in GFP + cell. It was also found that HIF-1α was not only distributed in the cytoplasm of MSCs but also concentrated in the peri-nucleus (Figure 1B).

### Effects of Ad-null-GFP and Ad-HIF-1α-GFP on MSCs

After transfection, cell viability and proliferation of each group were determined (Figure 1A IV). Viability and proliferation rates of ^Ad-HIF-1α^MSCs group were higher than other groups during the first two days after transfection (*P* <0.01), indicating that HIF-1α may improve the cell viability and proliferation *in vitro*. Meanwhile, viability and proliferation rates of the ^Ad-null^MSCs group 24 h after transfection decreased compared to the MSCs group (*P* <0.01), indicating that the transfection process might mildly affect the viability and proliferation of MSCs.

The representative photographs of MSC migration stained with DAPI were shown in Figure 1C. The migration was significantly increased in the ^Ad-HIF-1α^MSCs compared to other groups (*P* <0.01). These results demonstrated that HIF-1α can improve the cell viability, proliferation and motility of MSCs.

### Gene expression in the peri-infarcted regions of the heart

**Figure 2:**
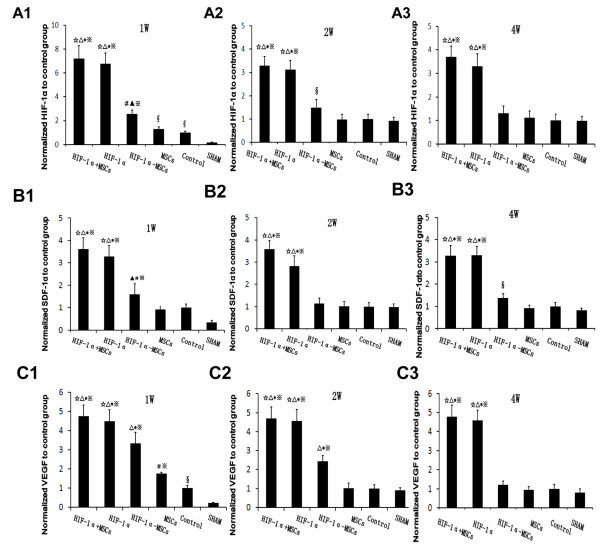
**The quantitative analysis of mRNA expression of HIF-1α, SDF-1α and VEGF. A1, A2, A3:** mRNA expression levels of hypoxia-inducible factor-1α (HIF-1α) in peri-infarcted region at one week, two weeks and four weeks after operation in each group, respectively. **B1, B2, B3:** mRNA expression levels of stromal cell-derived factor-1α (SDF-1α) in the peri-infarcted region at one week, two weeks and four weeks after operation in each group, respectively. **C1,C2, C3:** mRNA expression levels of vascular endothelial growth factor (VEGF) in the peri-infarcted region at one week, two weeks and four weeks after operation in each group, respectively. The expression levels were normalized to the Control group. ☆*P* <0.01 vs. HIF-1α- mesenchymal stem cells (MSCs) group, △*P* <0.01 vs. MSCs group, *P <0.01 vs. Control group, ※*P* <0.01 vs. SHAM group, ▲*P* <0.05 vs. MSCs group, **#***P* <0.05 vs. Control group, **§***P* <0.05 vs. SHAM group.

After the transplantation of MSCs and transfection of *HIF-1α*, we first evaluated the mRNA expression of *HIF-1α* in the peri-infarcted region of the heart via RT-PCR. *HIF-1α* mRNA expressions were significantly higher at one week, two weeks and four weeks post transfection in the HIF-1α + MSCs group and the HIF-1α group than other groups, indicating the successful transfection of the *HIF-1α* gene (Figure 2A1-A3). Specifically, at one week, two weeks and four weeks after operation, levels of *HIF-1α* mRNA in the HIF-1α + MSCs group increased for 6.4, 2.3 and 2.7 times over levels in the Control group, respectively. The HIF-1α-MSCs group showed slight increase in *HIF-1α* mRNA expression compared to the Control group at one week and two weeks after operation (Figure 2A1, A2). Although the Control group manifested significant increase in *HIF-1α* mRNA expression compared to the SHAM group at one week after operation (Figure 2A1), levels of *HIF-1α* mRNA were as low as the SHAM group (Figure 2A2, A3) at both two weeks and four weeks after infarction, indicating a temporary increase of HIF-1α due to acute ischemia after surgery.

We then evaluated two downstream genes whose transcriptional activities are directly modulated by *HIF-1α*, *SDF-1α* and *VEGF*. Similarly, both *SDF-1α* and *VEGF* mRNA levels were significantly higher at one week, two weeks and four weeks post transfection in HIF-1α + MSCs group and HIF-1α group than other groups (Figure 2B1-B3, C1-C3). Specifically, at one week, two weeks and four weeks after infarction, levels of *SDF-1α* mRNA in HIF-1α + MSCs group increased for 2.6, 2.6 and 2.3 times over levels in Control group, respectively (Figure 2B1-B3), while levels of *VEGF* mRNA increased for 3.8, 3.7 and 3.8 times (Figure 2C1-C3), suggesting that overexpressed *HIF-1α* increased the expression of *SDF-1α* and *VEGF*. The mRNA level of *VEGF* in the HIF-1α-MSCs and MSCs group increased for 2.3 and 0.5 times, respectively, over levels detected in Control group at one week after infarction (Figure 2C1). Although the Control group manifested a two-fold and three-fold increase in *SDF-1α* and *VEGF* mRNA expression compared to SHAM group at one week after operation (Figure 2B1, C1), by two weeks and four weeks after infarction, levels of *SDF-1α* and *VEGF* mRNA were as low as SHAM group, which is consistent with the expression pattern of *HIF-1α* (Figure 2B3, C2-C3).

### The cell engraftment in the recipient hearts

**Figure 3:**
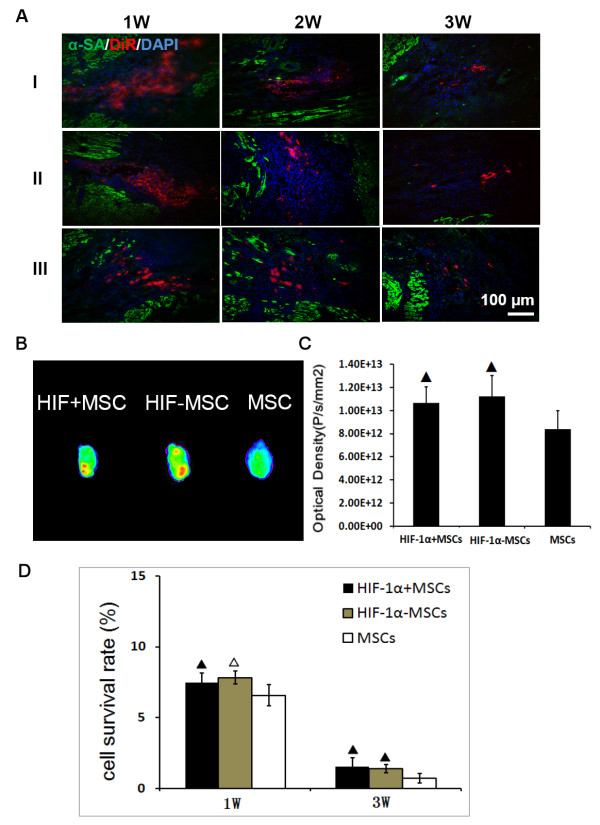
**Effects of HIF-1α on MSC engraftment in the border zone. A)** Engrafted mesenchymal stem cells (MSCs) stained with DiR (red) were detected one week, two weeks and three weeks after cell injection. The samples were stained with anti-α-SA antibody (green) and 4, 6-diamino-2-phenylindole (DAPI) (blue). **B**, **C)** Three weeks after cell injection, five hearts from three groups were harvested and imaged for detection of red fluorescence. The optical density (photon/s/mm^2^) from a fixed region of interest (ROI) was measured. **D)** Donor male cells persistent in the female hearts were detected by quantitative PCR for the *SRY* gene one week and three weeks after cell injection. ▲*P* <0.05 vs. MSCs group, △*P* <0.01 vs. MSCs group. HIF-1α, hypoxia-inducible factor-1α.

Frozen sections were detected under a fluorescence microscope. More engrafted MSCs, which were originally stained with DiR (red) before transplantation, were observed in HIF-1α + MSCs group and HIF-1α-MSCs group than that of MSCs group (Figure 3A). Likewise, fluorescence imaging revealed more red fluorescence in hearts from the HIF-1α + MSCs group and the HIF-1α-MSCs group (Figure 3B) compared to the MSCs group, indicating that exogeneous HIF-1α expression may improve the survival rate of MSCs. Optical density showed approximately a 0.26-fold and 0.33-fold increase in the HIF-1α + MSCs group and the HIF-1α-MSCs group compared to the MSCs group (Figure 3C). However, no difference was found between the HIF-1α + MSCs group and the HIF-1α-MSCs group.

To further quantitatively compare the survival rate of transplanted MSCs in the myocardium between different groups, quantitative PCR for the male-specific *SRY* gene was performed at one week and three weeks after cell injection. PCR results indicated that all of the three groups experienced a huge decrease of engrafted MSCs during the three weeks (Figure 3D). The survival rate of the transplanted MSCs declined from 7.4% to 1.5% in the HIF-1α + MSCs group, from 7.8% to 1.4% in the HIF-1α-MSCs group, and from 6.6% to 0.7% in the MSCs group. However, the HIF-1α + MSCs and HIF-1α-MSCs groups still exhibited enhanced cell engraftment relative to the MSCs group at both one week (7.4% vs. 7.8% vs. 6.6%, *P* = 0.001) and three weeks (1.5% vs. 1.4% vs. 0.7%, *P* <0.05) after cell injection (Figure 3D).

### The migration of MSCs into the infarction area

**Figure 4:**
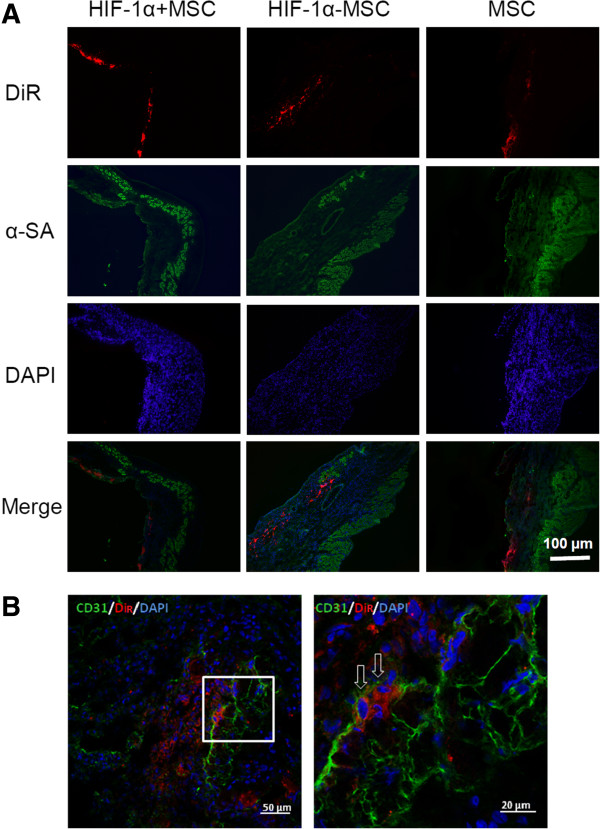
**Migration and differentiation of engrafted MSCs after cell injection. A)** Migration of mesenchymal stem cells (MSCs) at four weeks after cell injection from the border zone to infarcted region. Engrafted MSCs stained with DiR, which were injected originally in the peri-infarcted region, were detected in the infarcted region. The figures demonstrated MSCs engrafted in the infarcted region. **B)** Engrafted MSCs contribute to the angiogenesis three weeks after cell injection, in the peri-infarcted region of hearts of hypoxia-inducible factor-1α (HIF-1α) + MSCs group. Engrafted MSCs were stained with DiR (red) before cell injection. The arrow indicates CD31+ engrafted MSCs stained with DiR. Samples were stained with anti-CD31 antibody (green) and 4, 6-diamino-2-phenylindole (DAPI) (blue).

At three weeks after cell transplantation, hearts from six animals in each group were harvested and cryosectioned for histological analysis. Under a fluorescence microscope, engrafted MSCs were detected not only in the peri-infarcted region where cells were originally injected, but also widely distributed in the infarction region. More engrafted MSCs were detected in the infarction region of hearts from the HIF-1α + MSCs group compared to the HIF-1α-MSCs and MSCs groups (Figure 4A), indicating the migration of transplanted MSCs to the ischemic region of the infarcted heart can be enhanced by exogeneous HIF-1α expression.

Sections were then stained for CD31 and counter-stained with DAPI. Confocal microscopy was performed for detection of transplanted cells. It was detected that some engrafted MSCs were co-localized with the angiogenic marker CD31 (Figure 4B), suggesting that the transplanted cells may enhance angiogenisis.

### Vascular density measurement

**Figure 5:**
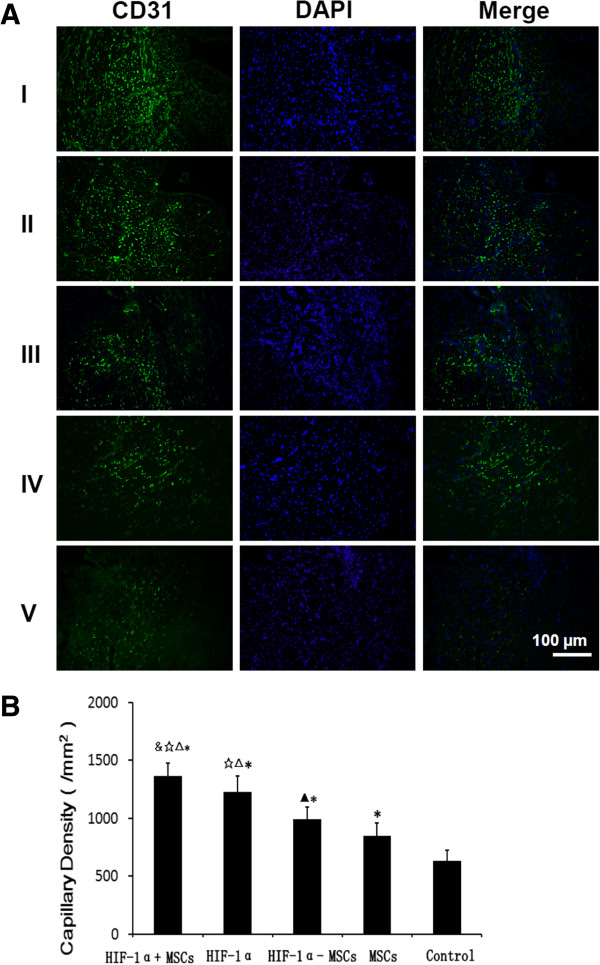
**Capillary densities at the peri-infarcted border zone in each group. A)** More capillary densities were detected in the hypoxia-inducible factor-1α (HIF-1α) + mesenchymal stem cells (MSCs) group than other groups. (I) HIF-1α + MSCs group, (II) HIF-1α group, (III) HIF-1α-MSCs group, (IV) MSCs group, (V) Control group. **B)** The number of CD-31-stained capillary was expressed as the number/mm^2^. &*P* <0.05 vs. HIF-1α group, ☆*P* <0.01 vs. HIF-1α-MSCs group, △*P* <0.01 vs. MSCs group, *P <0.01 vs. Control group, ▲*P* <0.05 vs. MSCs group.

**Figure 6:**
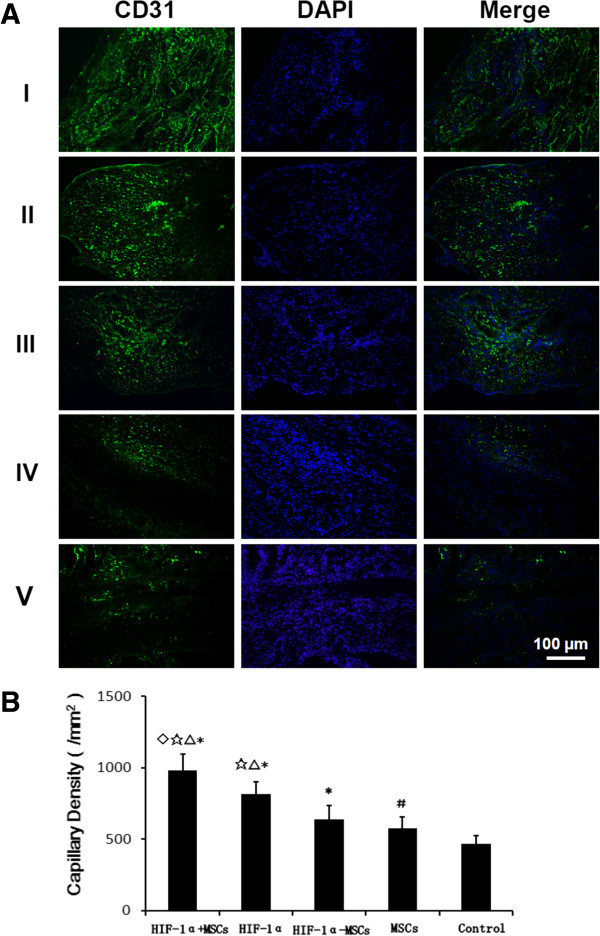
**Capillary densities in the infarction region in each group. A)** More capillary densities were detected in the hypoxia-inducible factor-1α (HIF-1α) + mesenchymal stem cells (MSCs) group than other groups. (I) HIF-1α + MSCs group, (II) HIF-1α group, (III) HIF-1α-MSCs group, (IV) MSCs group, (V) Control group. **B)** The number of the CD-31-stained capillary was expressed as the number/mm^2^. &*P* <0.01 vs. HIF-1α group, ☆*P* <0.01 vs. HIF-1α-MSCs group, △*P* <0.01 vs. MSCs group, *P <0.01 vs. Control group, #*P* <0.05 vs. Control group.

To assess the angiogenic effect of constitutive HIF-1α expression and MSC transplantation, we measured vascular density in the peri-infarcted (border zone) region adjacent to the infarction (Figure 5) and within the infarction region of the left ventricle (Figure 6) at four weeks after coronary ligation. We excised hearts and performed immunohistochemical staining with antibody against CD31 to detect endothelial cells. In the peri-infarcted area (Figure 5A, B), the capillary density observed in the hearts from HIF-1α + MSCs group (1,364 ± 128/mm^2^) was significantly higher than that of HIF-1α group (1,226 ± 140/mm^2^; *P* <0.05), HIF-1α-MSCs group (989 ± 110/mm^2^, *P <*0.01), MSCs group (849 ± 111/mm^2^; *P* <0.01), and Control group (630 ± 97/mm^2^; *P* <0.01). The capillary density of the HIF-1α group was significantly higher than the HIF-1α-MSCs, MSCs and Control groups (*P* <0.01). The capillary density of the HIF-1α-MSCs group was higher compared to the MSCs (*P* <0.05) and Control groups (*P* <0.01). Within the site of infarction (Figure 6A, B), the capillary density of the HIF-1α + MSCs group (978 ± 114/mm^2^) was also significantly higher than that of the HIF-1α (812 ± 91/mm^2^; *P* <0.01), the HIF-1α-MSCs (640 ± 94/mm^2^; *P* <0.01), the MSCs (573 ± 82/mm^2^; *P* <0.01), and the Control groups (469 ± 53/mm^2^; *P* <0.01). The capillary density of the HIF-1α group was significantly higher than the HIF-1α-MSCs, MSCs and Control groups (*P* <0.01). The capillary density of the HIF-1α-MSCs group was higher compared to the MSCs and Control groups (*P* <0.01). Grossly and microscopically, no angioma formation was observed in any treated animals or controls. The increased capillary density was mainly limited to the area around the infarcted and peri-infarcted area.

### Cell apoptosis in the infracted area

**Figure 7:**
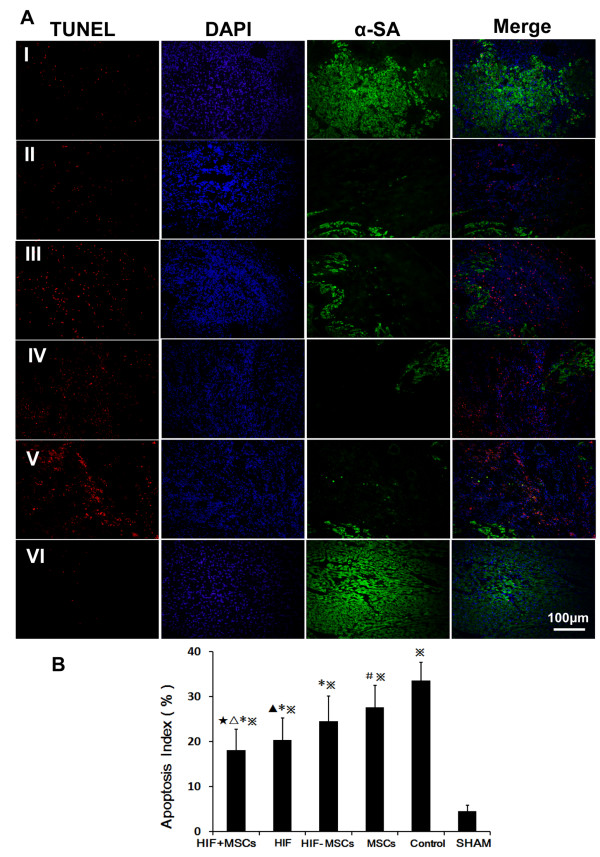
**Cardiomyocyte apoptosis in each group. A)** Hypoxia-inducible factor-1α (HIF-1α) and mesenchymal stem cell (MSC) intramyocardial injection decreased cardiomyocytes apoptosis one week after infarction. Apoptosis nuclei stained by terminal deoxynucleotidyl transferase dUTP nick end labeling (TUNEL) (red), total nuclei were labeled with 4, 6-diamino-2-phenylindole (DAPI) (blue). Cardiomyocytes were stained with anti-α-SA antibody (green). (I) HIF-1α + MSCs group, (II) HIF-1α group, (III) HIF-1α-MSCs group, (IV) MSCs group, (V) Control group, VI. SHAM. **B)** Apoptosis Index. ★*P* <0.05 vs. HIF-1α-MSCs group, △*P* <0.01 vs. MSCs group, *P <0.01 vs. Control group, ※*P* <0.01 vs. SHAM group, ▲*P* <0.05 vs. MSCs group, #*P* <0.05 vs. Control group.

Apoptotic extent was assessed via TUNEL staining. The number (as a percentage of the total) of cardiomyocytes showing apoptosis in the border zone was compared among groups (Figure 7, n = 5 in each group). The percentage of apoptotic cells was reduced in the HIF-1α + MSCs group (18.08 ± 4.59%) when compared to the HIF-1α-MSCs (24.43 ± 5.68%, *P* <0.05), MSCs (27.53 ± 4.90%, *P* <0.01) and Control groups (35.51 ± 4.03%, *P* <0.01). The HIF-1α group (20.36 ± 4.88%) showed decreased cell apoptosis compared to the MSCs (*P* <0.01) and Control groups (*P* <0.01). The HIF-1α-MSCs group showed less cell apoptosis when compared to the Control group (*P* <0.01).

### Cardiac performance after myocardial infarction

In the clinical setting, the prevention of progressive heart failure as a result of myocardial infarction is of great importance. To this end, we assessed the cardiac function after myocardial infarction one week, two weeks and four weeks after myocardial infarction by echocardiography. LVEF and FS evaluated by echocardiograph are shown in Table 2. At one week and two weeks after LAD ligation, markedly decreased LVEF and FS were detected with no significant difference among groups, corresponding to post-infarction myocardial failure. At four weeks after infarction, higher LVEF and FS were observed in the HIF-1α + MSCs group compared with the HIF-1α (*P* <0.05), HIF-1α-MSCs (*P* <0.05), MSCs (*P* <0.01) and Control groups (*P* <0.01), indicating that the combined HIF-1α and MSCs intramyocardial injection can significantly improve the cardiac function. No significant differences of LVEF and FS were observed among the HIF-1α, HIF-1α-MSCs, MSCs and Control groups.

**Table 2:** Assessment of LV function (EF and FS) by echocardiography

**Group**	**Baseline**	**1 W**	**2 W**	**4 W**
EF (%)				
Control	91.61 ± 5.79	32.16 ± 5.05	30.72 ± 3.19	25.87 ± 5.58
MSCs	90.51 ± 6.70	34.97 ± 5.87	32.23 ± 8.52	27.26 ± 6.38
HIF-1α-MSCs	91.07 ± 6.23	35.31 ± 7.46	33.10 ± 8.12	29.23 ± 8.63
HIF-1α	92.12 ± 5.54	35.57 ± 6.82	32.94 ± 7.38	30.99 ± 9.00
HIF-1α + MSCs	91.95 ± 6.71	37.18 ± 8.07	38.25 ± 5.72	40.96 ± 8.91^&★△*^
FS (%)				
Control	64.71 ± 2.28	16.48 ± 2.64	15.17 ± 1.80	12.60 ± 2.61
MSCs	63.64 ± 2.21	17.42 ± 3.20	16.51 ± 5.38	13.38 ± 4.52
HIF-1α-MSCs	64.07 ± 2.89	17.57 ± 4.09	16.64 ± 4.17	14.35 ± 4.07
HIF-1α	64.92 ± 2.94	17.86 ± 3.85	16.53 ± 3.91	15.62 ± 4.05
HIF-1α + MSCs	64.45 ± 3.15	18.88 ± 5.69	19.42 ± 3.27	21.04 ± 4.10^&★△*^

^&^*P* <0.05 vs. HIF-1α group, ^★^*P* <0.05 vs. HIF-1α-MSCs group, △*P* <0.01 vs. MSCs group, *P <0.01 vs. Control group. EF, ejection fraction; FS, fractional shortening; HIF-1α, hypoxia-inducible factor-1α; MSCs, mesenchymal stem cells.

## Discussion

Bone marrow-derived MSCs have become therapeutically important agents because of their multilineage potentials, immuno-modulatory properties and ability to localize specifically to injured sites, to reduce scar tissue formation and to increase neovascularization [4,40]. Although many MSC transplantation studies have shown beneficial effects in treating ischemic injury, it is currently limited by the poor engraftment of implanted MSCs due to the harsh microenvironment in the ischemic region [4,5]. Thus, additional strategy is required to enhance the therapeutic efficiency of cell therapy by improving cell homing, survival, engraftment and repair capacity of transplanted cells. One promising strategy may be the combination of cell and gene therapy.

Our results demonstrate that intramyocardial injection of *Ad-HIF-1α* combined with MSC transplantation was potent to promote angiogenesis, and led to improved cardiac performance after myocardial infarction in the rats. Our study also showed that overexpression of *HIF-1α* after *Ad-HIF-1α* transfection allows for supranormal levels of *HIF-1α* mRNA in peri-infarcted myocardium. Consistent with previous studies [29,33], we found that in the SHAM group, without hypoxia, the mRNA level of *HIF-1α* was extremely low (Figure 2A1-A3). While in the Control group, which presented the endogenous *HIF-1α* expression under hypoxia conditions, the mRNA level of *HIF-1α* was significantly increased. The mRNA levels of *HIF-1α* in the HIF-1α + MSCs and HIF-1α groups were expressed at much higher levels than that of the MSCs and Control groups due to the exogeneous *HIF-1α* expression. However, the *HIF-1α* mRNA was much lower in the HIF-1α-MSCs group compared to the HIF-1α + MSCs and HIF-1α groups. The *HIF-1α* expression level in the HIF-1α-MSC group depends on the numbers of engrafted MSCs, which have very low survival rates as evidenced by our data. In contrast, the injected virus could transfect any cell type in the infarcted area, which may explain the observed differences of *HIF-1α* expressions among different groups.

We found that the *SDF-1α* mRNA expressions were also significantly increased in the HIF-1α + MSCs and HIF-1α groups than in that of other groups (Figure 2B1-B3). Previous research found that the SDF-1α/CXCR4 axis mediated the migration and homing of bone marrow-derived cells and endothelial progenitor cells *in vivo*[30,41-43]. Consistent with these findings, we found that more MSCs survived and engrafted in the infarcted hearts in the HIF-1α + MSCs and HIF-1α-MSCs groups than in the MSCs group. Enhanced angiogenesis, better blood flow and the beneficial effect of several cytokines may contribute to the improvement of the ability of MSCs to survive in hypoxic environments, the migration to the ischemic fibrotic tissue from the border zone, and the angiogenesis in the ischemic area. Since more MSCs survived in the infarcted hearts in the HIF-1α + MSCs and HIF-1α-MSCs groups, we speculated that the combined therapy may further enhance the angiogenesis at the peri-infarcted and infarct regions compared to the HIF-1α group, partly due to the MSC-dependent paracrine mechanism.

The mRNA levels of *VEGF*, regulated directly by *HIF-1α*, were expressed at much higher level in the HIF-1α + MSCs and HIF-1α groups compared to other groups (Figure 2C1-C3). It partly explained why a marked increase in capillary density in the peri-infarcted and infarcted regions was observed in these two groups. Sufficient blood flow as a result of increased blood vessel formation might be instrumental in preventing the loss of cardiomyocytes in these zones over time, preservation of contractility in the border zone adjacent to the infarct, and suppression of post-infarction cardiac failure during left ventricular remodeling [29].

Our findings suggest that myocardial deterioration after infarction in the HIF + MSCs group may be limited not only as a result of stimulation of angiogenesis through a VEGF-related pathway, but also through additional HIF-1α-mediated local adaptations to low oxygen tension, and significantly improved microenvironment and increased survival, engraftment and repair ability of MSCs. Furthermore, our research demonstrated that the HIF + MSCs group showed better capacity for cardiac repair in terms of the expression of certain important cytokines, such as VEGF,、SDF-1α, pro-angiogenesis, anti-apoptosis and restoration of heart function than the HIF-MSCs group. Our report suggests the possibility of using the combination of cell and gene therapy to improve the cardiac repair, without reported side effects, such as fragile and immature vessels and angioma formation [44,45].

Additionally, our study demonstrated that HIF-1α + MSC treatment was superior to *HIF-1α* transfection alone in terms of pro-angiogenesis, anti-apoptosis and the capacity for cardiac function repair. Consistent with previous references [46,47], our study showed that MSCs contribute to the angiogenesis and anti-apoptosis, which may be partly due to the MSC-dependent paracrine mechanism and their potential for trans-differentiation [48-51].

Although we demonstrated that the cell survival rate was higher in the HIF-1α + MSCs and HIF-1α-MSCs groups when compared to the MSCs group, the engraftment of cells decreased significantly in all three groups. One hypothesis that may explain the drop-off in engraftment and survival rate is that the exogenous *HIF-1α* may have been degraded or metabolized. In our future study, Western blotting will be used to track the amount of HIF-1α available to cells over time *in vivo* in order to clarify the role of HIF-1α in cardiac repair. Furthermore, several studies recently demonstrated encouraging results via the use of biomaterials, such as a dendrimer-type bio-reducible polymer or facial amphipathic bile acid-conjugated polyethyleneimine, to aid the localization of therapeutic genes to the target cells, thus improving the transfection efficiency and enhancing the cardiac repair [52,53]. Application of such biomaterials in gene therapy holds promise as a potential novel therapy for the treatment of myocardial ischemia and infarction.

So far, the low engraftment of transplanted cells is still the major obstacle to the wide application of stem cell transplantation to treat MI. A series of studies have demonstrated several potential strategies to optimize stem cell engraftment, such as using tissue-engineered collagen-based scaffolds to provide a suitable microenvironment to support cell attachment and proliferation [54,55], or using dynamic three-dimensional culture techniques to enhance MSCs’ properties and increase therapeutic potential [56,57]. Recently, our team found that magnetic targeting enhanced the retention of magnetized stem cell in a rat model of myocardial infarction, suggesting that magnetic targeting offers new perspectives for enhancing the cell retention and subsequent functional benefit in heart diseases [58]. And in our future study, we plan to combine physics and biological methods to optimize the engraftment rate.

## Conclusions

We demonstrated that intramyocardial transfection of *HIF-1α* and co-transplantation of mesenchymal stem cells enhanced cardiac repair in an experimental model of MI, in correlation with increased ability to induce angiogenesis, reduced apoptosis, and increased survival and engraftment of MSCs, thus having transformative impact on the treatment of patients suffering from severe myocardial ischemia.

## Abbreviations

Ang: Angiopoietin; CCK-8: Cell Counting Kit-8; DAPI: 4, 6-diamino-2-phenylindole; DiR: 1, 1-dioctadecyl-3,3,3,3-tetramethyl indotricarbocyanine iodide; DMEM: Dulbecco's Modified Eagle Medium; FBS: Fetal bovine serum; FITC: Fluorescein isothiocyanate conjugated; FS: Fractional shortening; GFP: Green fluorescent protein; HIF-1α: Hypoxia-inducible factor-1α; iNOS: Nitric oxide synthase; LAD: Anterior descending coronary artery; LVEF: Left ventricular ejection fraction; MACE: Major adverse cardiac events; MI: Myocardial infarction; MSCs: Mesenchymal stem cells; PBS: Phosphate-buffered saline; PFU: Plate forming unit; SDF-1α: Stromal cell-derived factor-1α; TUNEL: Terminal deoxynucleotidyl transferase dUTP nick end labeling; VEGF: Vascular endothelial growth factor; α-SA: α-sarcomeric actinin.

## Competing interests

The authors have no financial conflicts of interests.

## Authors’ contributions

BQH participated in conception and design, data collection and analysis, and manuscript writing. JYQ contributed to the conception and design, data collection and analysis, and critical revision of the manuscript. JYM and ZYH participated in data collection and analysis, and critical revision of the manuscript. UCG, YLS and XYC contributed to data collection and analysis. AJS participated in the conception and design, obtaining financial support, and manuscript writing. JBG participated in conception and design, data collection and analysis, and manuscript writing. HZC contributed to data collection and analysis, and manuscript writing. All authors read and approved the final manuscript.

## Supplementary Material

Additional file 1 **Identification and differentiation assay of MSCs. A**. Identification of MSCs. MSCs uniformly expressed CD105 and CD166, but not CD45 or CD34. **B**. Differentiation assay showed the differentiation potentials of the isolated MSCs into osteoblasts (alizarin red), adipocytes (oiled red) and chondrocytes (alcian blue). Click here for file
