# Supplementary figures and images for: Myocardial transfection of hypoxia-inducible factor-1α and co-transplantation of mesenchymal stem cells enhance cardiac repair in rats with experimental myocardial infarction
Source: Stem Cell Res Ther. 2014 Feb 7;5(1):22. doi: 10.1186/scrt410 (PMC4055118; doi:10.1186/scrt410)

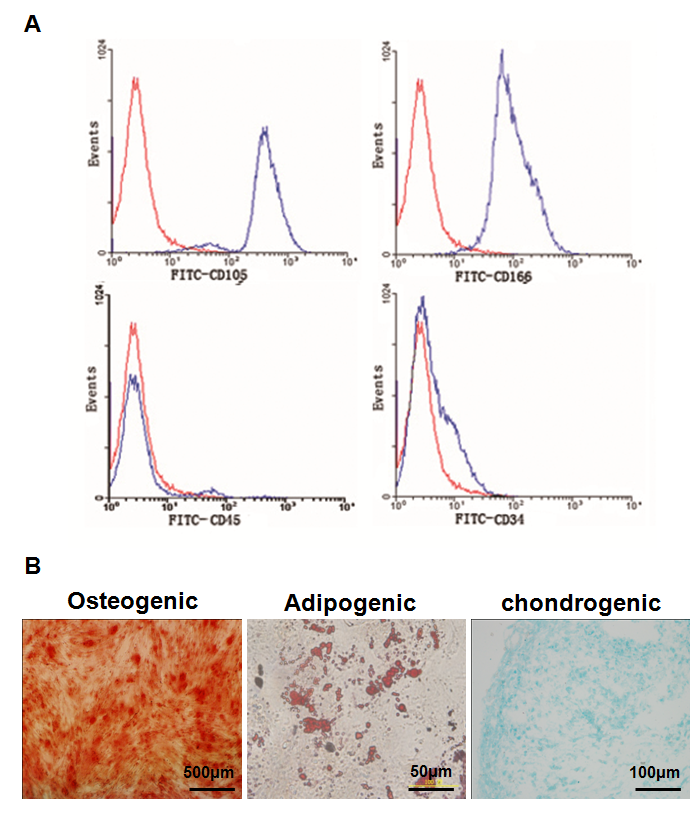

Supplement: Additional file 1 — Identification and differentiation assay of MSCs. A. Identification of MSCs. MSCs uniformly expressed CD105 and CD166, but not CD45 or CD34. B. Differentiation assay showed the differentiation potentials of the isolated MSCs into osteoblasts (alizarin red), adipocytes (oiled red) and chondrocytes (alcian blue). [file scrt410-S1.tiff]
